# A Portable Real-Time Quantitative Colorimetric LAMP Workflow for Early In-Situ Detection of *Botrytis cinerea* in Tomato Plants

**DOI:** 10.3390/plants15213462

**Published:** 2026-11-06

**Authors:** Stefanos K. Soultatos, Maria Megariti, Nikolaos E. Krasagakis, George Papadakis, Stella Chatziioannidou, Despoina Kourougkiaouri, Electra Gizeli, Emmanouil A. Markakis

**Affiliations:** 1 Department of Agriculture, School of Agricultural Sciences, Hellenic Mediterranean University, Stavromenos, 71410 Heraklion, Greece; ddk279@edu.hmu.gr (S.K.S.); nikos.krasagakis@yahoo.gr (N.E.K.); 2 Department of Biology, University of Crete, Voutes, 70013 Heraklion, Greece; maria_megariti@imbb.forth.gr (M.M.); gizeli@imbb.forth.gr (E.G.); 3 Institute of Molecular Biology and Biotechnology, Foundation for Research and Technology-Hellas, 100 N. Plastira Str., 70013 Heraklion, Greece; 4 BIOPIX DNA TECHNOLOGY S.A., Science and Technology Park of Crete, 100 N. Plastira Str., 70013 Heraklion, Greece; gpapadakis@biopix-t.com (G.P.); schatziioannidou@biopix-t.com (S.C.);

**Keywords:** point-of-care diagnostics, latent infection, grey mould, *Solanum lycopersicum*, isothermal amplification, crude DNA extraction

## Abstract

In the present study, a novel method was developed and validated for the early, rapid and on-site detection of *Botrytis cinerea* in crude tomato samples using a loop-mediated isothermal amplification (LAMP) assay adapted for the portable device PEBBLE. Previously reported LAMP primers (targeting the IGS region of *rDNA* gene) were utilized and optimized in terms of concentration for this workflow, and the sensitivity, specificity and accuracy of the developed method were determined (97.96%, 100% and 98.70%, respectively). The LAMP assay, combined with the portable device, enabled real-time quantitative colorimetric (qcLAMP) detection of *B. cinerea* in less than 30 min. The limit of detection (LoD) of the qcLAMP assay was 1 pg genomic DNA (≈20 genome copies) per reaction, whereas conventional PCR (utilized as the gold standard method) detected no less than 100 pg of genomic DNA per reaction. In terms of *B. cinerea* infective propagules, qcLAMP could detect as few as 10^2^ spores in both pure and crude DNA samples extracted from artificially inoculated tomato leaves, whereas PCR detected no fewer than 10^4^ spores in pure DNA samples, failing to achieve detection in crude extracts. In in vivo bioassays, qcLAMP successfully detected *B. cinerea* immediately following spore deposition on leaf tissues (0 h post-inoculation) in both pure and crude DNA extracts. In contrast, conventional PCR was successful only in pure DNA samples from leaves collected 2 days post-inoculation (dpi) and in crude extracts at 3 dpi, when symptoms were already evident. Evaluations of in-field samples demonstrated that qcLAMP detected *B. cinerea* in 100% of crude extracts from symptomatic tissues and in 45% of asymptomatic (latently infected) samples. Conversely, conventional PCR failed to detect the pathogen in crude extracts, regardless of the disease status (symptomatic or asymptomatic). The developed method can serve as a rapid, reliable, and user-friendly tool for the early detection of *B. cinerea* in the field, enabling prompt assessment of inoculum pressure, thereby facilitating timely management interventions.

## 1. Introduction

*Botrytis cinerea* (teleomorph *Botryotinia fuckeliana*) is a widespread plant pathogenic fungus causing gray mould to a wide range of economically important crops cultivated under greenhouse and open-field conditions [1]. Its ability to persist as airborne and soilborne inoculum, colonize and infect wounded plant tissues rapidly under conductive environmental conditions, and develop genetic resistance to fungicides, makes it a consistent threat throughout the entire growing season. In tomato (*Solanum lycopersicum*), one of the most economically important hosts of the pathogen, the fungus attacks tissues of potentially all above-ground plant organs, causing severe losses at the pre- and the post-harvest levels, with the latter being driven by latent infections that remain undetected in the field [1,2].

Early detection of the pathogen is crucial, as disease symptoms often manifest only after infection is well established, thereby limiting the efficacy of management strategies. In this respect, several methods have been developed for the detection and quantification of *B. cinerea*, in planta. These include molecular-based techniques like polymerase chain reaction (PCR) [3], real time PCR [4,5], loop-mediated isothermal amplification (LAMP) [6,7], as well as plate-trapped antigen enzyme-linked immunosorbent [8] and microfluidic chip [9] assays. Furthermore, protocols have been established to detect and differentiate *B. cinerea* from other closely related species such as *B. fabae* and *B. squamosa* in broad bean [3] or *B. fragariae* in strawberry [10]. Conventional diagnostic approaches, such as laboratory-based molecular assays or culture-dependent methods, although accurate, are time-consuming, require specialized equipment, thorough sample manipulation and trained personnel, making them unsuitable for rapid in-field decision making [11].

To overcome these limitations, there is growing interest in developing portable diagnostic technologies that enable fast, sensitive and accurate detection of plant pathogens directly in the field [12]. Among these, loop-mediated isothermal amplification (LAMP) has emerged as a leading platform due to its high specificity, tolerance to crude sample inhibitors, and minimal equipment requirements [13]. LAMP, first described by Notomi et al. [14], is an isothermal nucleic acid amplification technique that employs two to three pairs of primers targeting specific DNA sequences together with a *Bst* DNA polymerase possessing high strand displacement activity, allowing rapid and efficient DNA amplification at a constant temperature of approximately 65 °C. Despite these considerable advantages, and the development of LAMP assays for several plant pathogens including fungi [15,16,17], oomycetes [18], bacteria [19,20,21], phytoplasmas [22,23], and viruses/viroids [24,25,26], the widespread adoption of this technique for routine field diagnostics in crop protection remains limited. This is largely attributable to the need for integrated diagnostic platforms that combine robust assay design, simplified sample preparation and portable detection systems suitable for on-site applications. In addition, comprehensive analytical and diagnostic validation is required to establish key performance characteristics, including sensitivity, specificity, reproducibility, and diagnostic accuracy, relative to conventional detection methods. Equally important is the development of appropriate sampling protocols tailored to biology, spatial distribution, and infection dynamics of the target pathogen, as these factors critically influence the reliability and accuracy of disease detection.

To address these gaps, the main objectives of the present study were to validate and apply an integrated real-time quantitative colorimetric LAMP (qcLAMP) diagnostic workflow for the rapid and early detection of *B. cinerea* in tomato plants. The distinct novelty of this methodology lies in the incorporation of established IGS-targeting primer sequences, (originally reported by Tomlinson et al. [6]), which were explicitly optimized in concentration to match a novel reaction chemistry and detection configuration. This system combines two technologies developed by BIOPIX DNA TECHNOLOGY: the Universal Mastermix, comprising a proprietary pre-optimized amplification formulation, and the portable PEBBLE^®^ diagnostic platform for real-time monitoring. Coupled with an ultra-simplified, buffer-free, water-based crude extraction protocol, this integrated system eliminates the need for laboratory infrastructure, enabling for the first time true on-site, field-based quantitative colorimetric (qcLAMP) analysis directly inside greenhouses or open fields. Crucially, to demonstrate its real-world applicability, the developed diagnostic platform has been robustly evaluated and validated using naturally infected plant samples, collected from a commercial greenhouse, ensuring its performance under authentic field pressure conditions. This provides a rapid (<30 min) and highly sensitive tool for detecting both symptomatic and latent (asymptomatic) *B. cinerea* infections, thereby facilitating timely and sustainable crop protection interventions.

## 2. Results

### 2.1. Sensitivity, Specificity, Accuracy and Detection Limit of the Developed qcLAMP Assay in Pure DNA Samples

**Figure 1:**
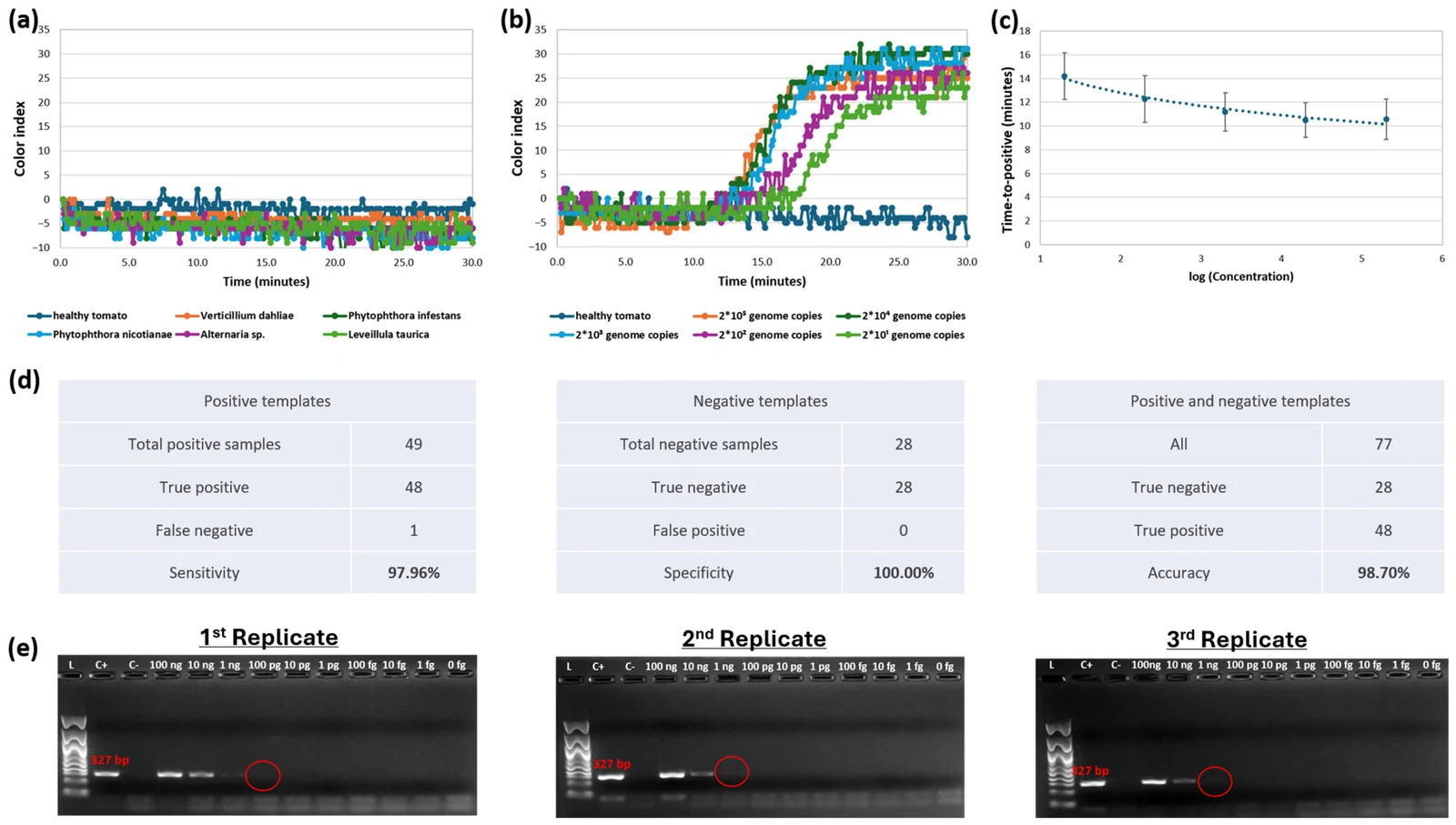
Sensitivity, specificity and accuracy assessment of the qcLAMP assay for *B. cinerea* detection. (**a**) Specificity analysis of the qcLAMP using representative DNA templates (1 ng/μL per reaction) from non-target microorganisms affecting tomato, as well as healthy tomato DNA; (**b**) Analysis of a 10-fold serial dilution of *B. cinerea* genomic DNA (ranging from 10 ng to 1 pg per reaction, corresponding to 2 × 10^5^ to 2 × 10^1^ genome copies); (**c**) Sensitivity curve of the qcLAMP assay; (**d**) Analytical performance metrics (sensitivity, specificity, and accuracy) of the test; (**e**) Replicated conventional PCR amplification assays of a 10-fold genomic DNA dilution series, generating a *B. cinerea*-specific 327-bp amplicon; red circles indicate the lowest amount of DNA that was detected in each replicate.

A total of 28 negative samples were analyzed (at a standard concentration of 1 ng/μL each), all of which were correctly identified as true negatives, corresponding to 100% specificity (Figure 1a,d). For the sensitivity evaluation, 49 positive samples (*B. cinerea* DNA) were tested across various template concentrations ranging from 10 ng/μL to 1 pg/μL (equivalent to ≈2 × 10^5^ to 2 × 10^1^ genome copies/μL); 48 were detected as true positives and 1 as a false negative, resulting in a sensitivity of 97.96% (Figure 1d). Additionally, to create the sensitivity curve, three independent runs using serial dilutions of the *B. cinerea* DNA template were performed. Figure 1b, illustrates representative curves of the serially diluted samples, while Figure 1c depicts final sensitivity curve, indicating that at higher template concentrations, the time-to-positive values exhibited no significant variation as they reached a plateau. Overall, 77 DNA templates were analyzed (49 positive and 28 negative samples), yielding an overall diagnostic accuracy of 98.70% for the extracted samples (Figure 1d). The lowest concentration of *B. cinerea* DNA consistently detected by the novel qcLAMP assay was 1 pg per reaction (≈20 genome copies), which is at least two orders of magnitude lower than the limit of detection achieved by conventional PCR (which ranged from 100 pg to 1 ng of DNA per reaction) (Figure 1b,e).

### 2.2. Comparison of TPS Buffer and Water-Based Crude DNA Extraction for qcLAMP Assays

**Figure 2:**
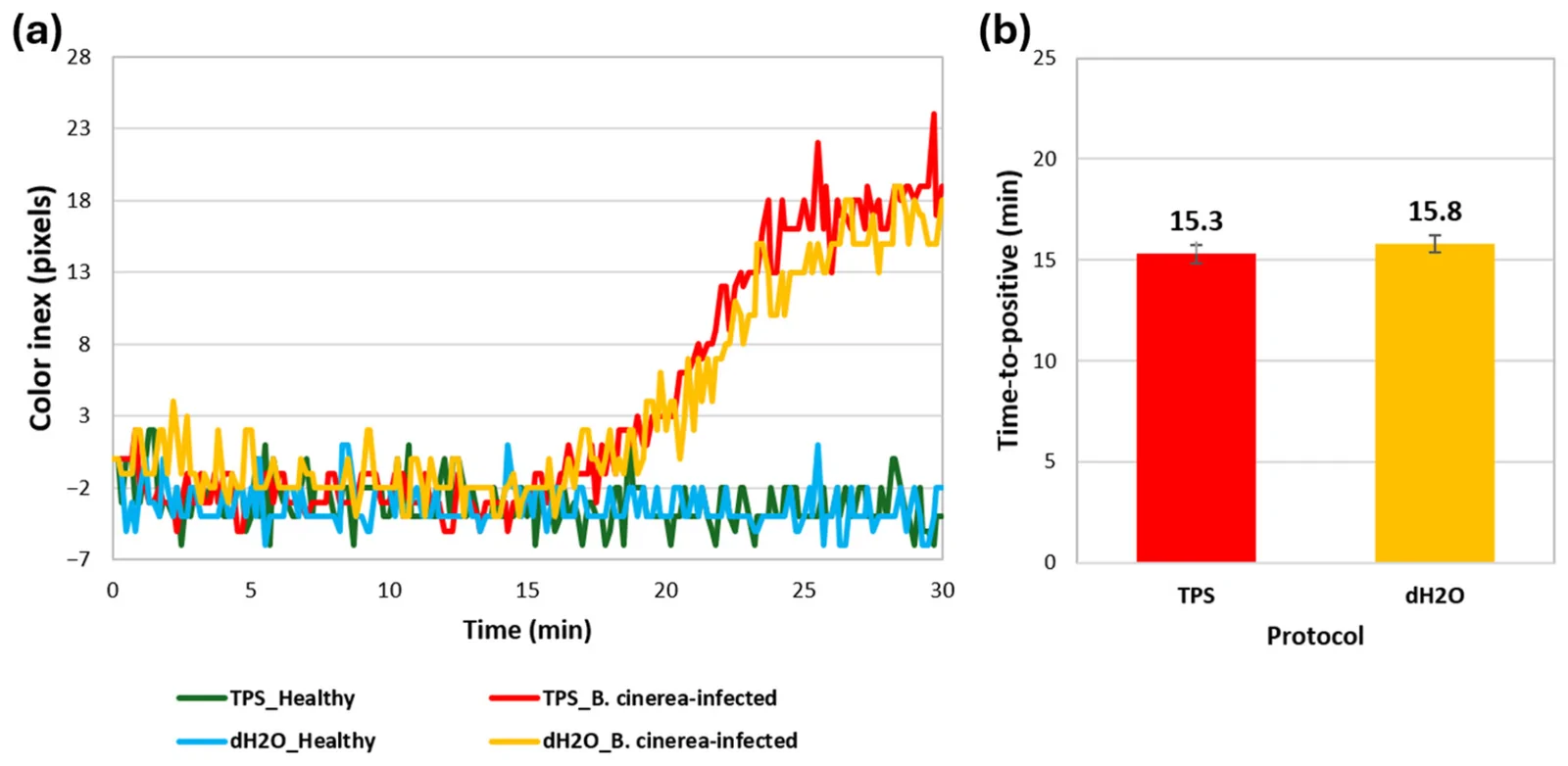
Comparative qcLAMP analysis of representative samples from *B. cinerea*-infected and healthy tomato plants, processed using the TPS buffer and the simplified water-based protocol. (**a**) Real-time colorimetric profiling demonstrating positive and negative detection of *B. cinerea*; (**b**) Mean time-to-positive values and standard deviation estimated across three independent replicates. No amplification was observed in samples from healthy plants.

The qcLAMP analysis of symptomatic leaf samples collected from *B. cinerea*-infected plants revealed no significant differences in terms of real-time colorimetric profiling and mean time-to-positive values, between the TPS buffer and dH_2_O treatments (Figure 2a,b). Furthermore, *B. cinerea* was not detected in samples obtained from healthy plants, processed with either extraction protocol. The presence or absence of the pathogen in all samples was verified using standard detection procedures (Cetyltetramethyl ammonium bromide (CTAB) DNA extraction method followed by qcLAMP and conventional PCR assays, alongside microscopic observations of fungal fructifications). Consequently, the simplified water-based protocol was adopted for the preparation of plant crude extracts in all subsequent experiments.

### 2.3. Effect of B. cinerea Inoculum Density on Grey Mould Symptom Severity

**Figure 3:**
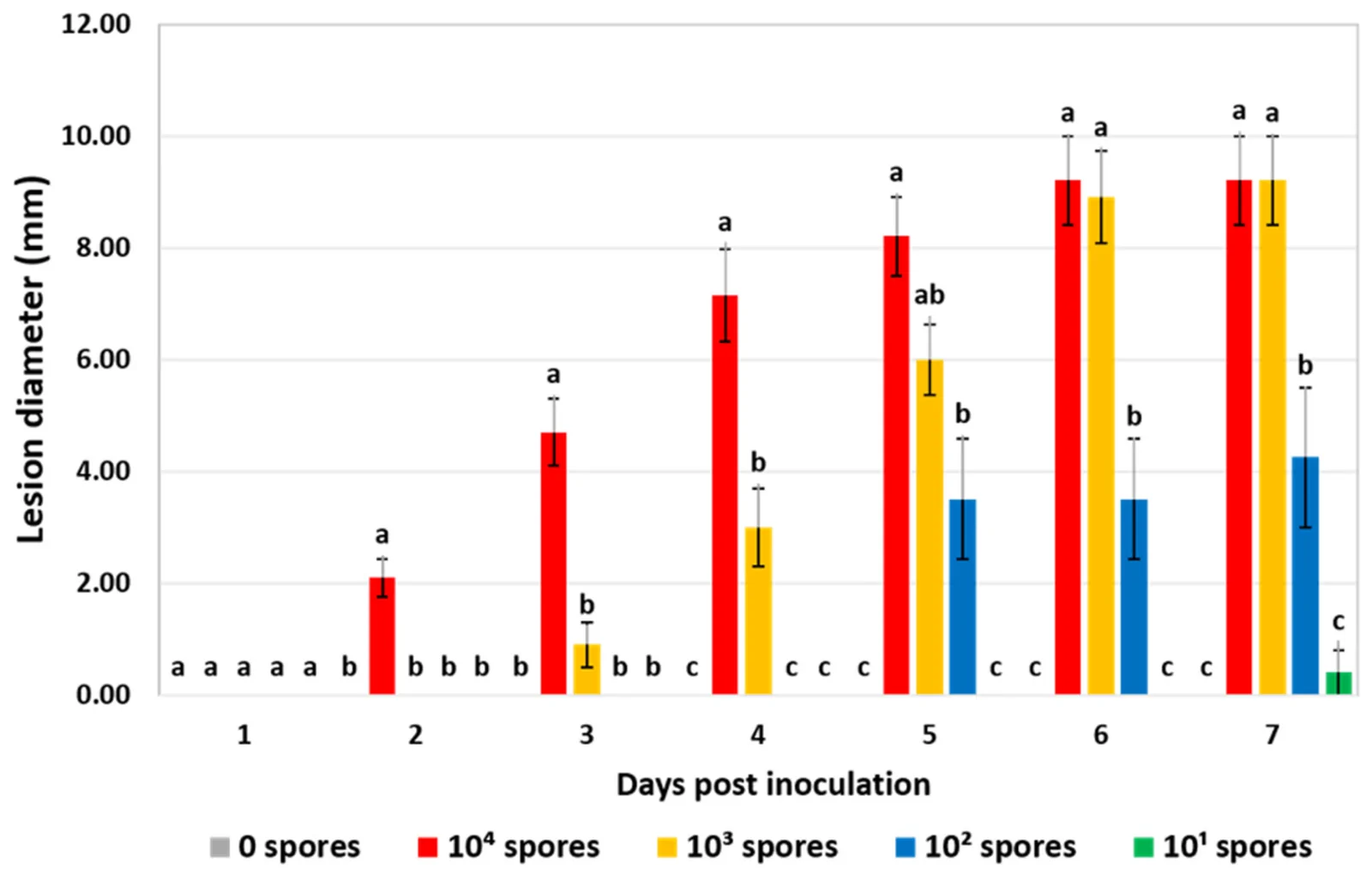
Symptom severity in leaf discs inoculated by depositing a 10-μL drop of 10^4^, 10^3^, 10^2^, 10^1^, or 0 spores of *B. cinerea* (isolate B.14.9) per disc, at daily intervals from 1 to 7 days post-inoculation. Within each observation time point, columns followed by the same letter are not significantly different according to Tukey’s HSD test (*p* ≤ 0.05). Each column represents the mean of 10 leaf discs, and vertical bars indicate standard errors.

Symptom severity data (lesion diameter) indicated that apart from the lowest inoculum level (10^1^ spores), all the other tested loads (10^2^, 10^3^, and 10^4^ spores) of *B. cinerea* (isolate B.14.9) infected leaf discs significantly, compared to the mock-inoculated control (0 spores). At 7 days post-inoculation (dpi) the final lesion diameter values were 9.20, 9.20, 4.25, 0.40 and 0.00 mm for 10^4^, 10^3^, 10^2^, 10^1^, and 0 spores, respectively, (Figure 3). Among these, the density of 10^3^ spores was selected as the optimal inoculum density. Crucially, the one-day delay in symptom onset (3 dpi) compared to the highest inoculum density (10^4^ spores) was highly advantageous as it expanded the span for presymptomatic monitoring while still achieving a steady disease progression and an identical final disease severity. Therefore, this inoculum density was selected and applied in the following experiments focusing on presymptomatic detection.

### 2.4. Sensitivity of qcLAMP for the Detection of B. cinerea Propagules in Crude Extracts

The results of the PCR and qcLAMP analyses of purified DNA and crude extracts obtained from leaf discs inoculated with varying *B. cinerea* spore densities (infective propagules) are presented in Table 1. The qcLAMP assay consistently detected as few as 10^2^ spores in purified DNA samples, as well as in two out of the three replicate crude extracts. However, time-to-positive values were significantly higher in the latter sample type. In contrast, the conventional PCR assay detected 10^5^ and 10^4^ spores exclusively in purified DNA samples. PCR failed to detect pathogen propagules ranging from 10^3^ down to 0 spores in purified DNA samples and yielded negative results for all spore concentrations in crude extracts.

### 2.5. Efficacy of qcLAMP for the Presymptomatic Detection of B. cinerea

**Figure 4:**
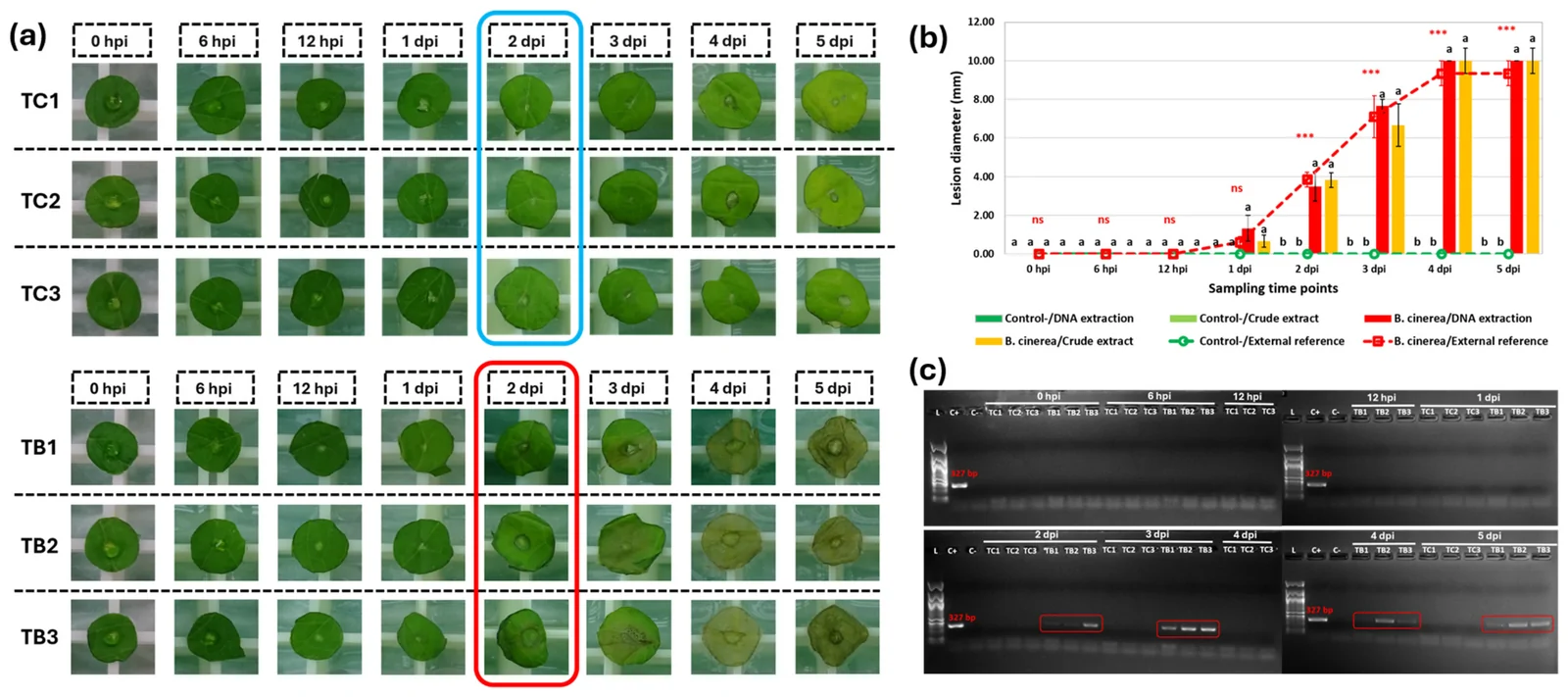
A leaf disc setup for the evaluation of qcLAMP assay efficiency for pre- and post-symptomatic detection of *B. cinerea*, compared with conventional PCR. (**a**) Symptom development over time in tomato leaf discs inoculated with a 10-μL drop containing 10^3^ *B. cinerea* spores (designated as TB1, TB2 and TB3) or sterilized distilled water (designated as TC1, TC2 and TC3), at 0, 6, 12 h post-inoculation (hpi) and 1, 2, 3, 4, 5 days post-inoculation (dpi). The red box highlights the onset of visible symptoms in inoculated discs at 2 dpi, while the blue box indicates the corresponding healthy control discs; (**b**) Disease progression (lesion diameter) in inoculated leaf discs sampled for either DNA extraction (red columns) or crude extracts (yellow columns), alongside external reference discs (red and green lines) monitored to track standard symptom development. Within each time point, columns followed by the same letter are not significantly different according to Tukey’s HSD test (*p* ≤ 0.05, n = 3). Within each time point, asterisks ‘***’ and ‘ns’ above markers, indicate highly significant (*p* ≤ 0.001) and non-significant differences, between inoculated and mock-inoculated discs, respectively, according to the *t* test (n = 10). Vertical bars indicate standard errors of the mean; (**c**) Conventional PCR detection of *B. cinerea* in purified DNA samples extracted from leaf discs, sampled across the identical time points (0 hpi to 5 dpi). Red boxes highlight positive detection indicated by the target-specific 327-bp amplicon band.

Practically, no symptoms were observed up to 1 dpi, in the inoculated leaf discs used for either DNA extraction or crude extracts, nor in those serving as external references to monitor disease progression (Figure 4a,b, Table 2). The minor symptoms recorded at 1 dpi reflected a faint, ring-like lesion (≤2 mm in diameter) around the inoculation in only a few discs, showing no significant differences between artificially inoculated and mock-inoculated controls (Figure 4b, Table 2). The onset of clear symptom development was observed from 2 dpi onwards, with disease severity increasing progressively and reaching a plateau at 4 dpi (Figure 4a,b). In contrast, control discs remained asymptomatic throughout the experiment period.

In parallel, the conventional PCR assay detected *B. cinerea* in purified DNA samples extracted from inoculated discs no earlier than 2 dpi, coinciding with the onset of visible symptoms. Positive detection was consistently achieved in all purified DNA samples collected at subsequent time points (Figure 4c, Table 2). In contrast, PCR amplification using crude extracts yielded positive *B. cinerea* detection in all 3 biological replicates exclusively at 3 dpi. Conversely, the qcLAMP assay successfully detected the target pathogen across all sampling time points, regardless of the template preparation method (purified DNA or crude extract) or tissue symptomatology (symptomatic or asymptomatic) (Table 2). However, time-to-positive values were significantly lower for purified DNA samples (ranging from 11 min to 16.5 min) compared to crude extracts (ranging from 11.3 min to 28.0 min) (Table 2). Notably, qcLAMP also yielded positive detection in three purified DNA samples from mock-inoculated discs collected at 12 hpi (1st replicate), 2 dpi (3rd replicate) and 3 dpi (2nd replicate), with corresponding time-to positive values of 20.3 min, 17.8 min and 16.7 min (Table 2). These positive reactions in mock-inoculated control likely reflect natural latent infections pre-existing in the greenhouse-grown plant material prior to excision, rather than experimental cross-contamination, a phenomenon further supported by our field survey data.

### 2.6. Comparative Diagnostic Efficacy of qcLAMP for B. cinerea Detection in Field Samples

The comparative detection performance of conventional PCR and qcLAMP using purified DNA and crude extracts from apparently healthy and *B. cinerea*-infected, greenhouse-collected tomato leaves is illustrated in Table 3. The conventional PCR detected *B. cinerea* in 19 out of 20 purified DNA samples (95% detection rate) extracted from diseased leaves, whereas no detection was achieved in apparently healthy samples. However, PCR amplification did not detect *B. cinerea* in crude extracts derived from either asymptomatic or symptomatic leaf tissues (Table 3). In contrast, the qcLAMP assay successfully detected the target pathogen in all purified DNA or crude extract samples derived from diseased tissues that were analyzed (100%) (Table 3). Interestingly, qcLAMP detected *B. cinerea* in 2 out of 20 purified DNA samples (10%) as well as in 9 out of 20 crude extract samples (45%) derived from visibly healthy tissues. Overall, time-to-positive values were significantly lower for purified DNA samples (ranging from 11.7 min to 23.2 min) compared to crude extracts (ranging from 11.0 min to 30.0 min) (Table 3).

## 3. Discussion

Early detection of plant pathogens is paramount for the timely implementation of disease management practices, which significantly enhances the effectiveness of control measures. Crucial to this approach is the development of robust sampling protocols tailored to biology, spatial distribution and infection dynamics of the target pathogen, as those factors critically influence diagnostic reliability and accuracy. This requirement is particularly evident for *B. cinerea*, an exemplary polycyclic fungal pathogen characterized by an extraordinary capacity for sporulation, rapid multiplication, and high genetic plasticity [1]. The latter drives fitness mutations that frequently complicate disease management [2]. The pathogen produces abundant airborne conidia capable of rapid dispersal, leading to severe infections across a wide range of economically important crops. Consequently, there is an urgent need for rapid, on-site, reliable and affordable diagnostic tools that can detect *B. cinerea* at early stages, especially outside conventional laboratory settings, to mitigate devastating yield losses. In the present study, a novel LAMP-based method was developed and validated for the early, rapid, and on-site detection of *B. cinerea* directly from crude tomato samples. This method incorporates the evaluation of a previously established LAMP assay, optimized for colorimetric reaction formulation, alongside a fast extraction protocol and the recently developed portable diagnostic device PEBBLE^®^ [27], enabling analysis outside laboratory facilities.

Beyond the initial in silico analyses, specificity testing using purified DNA from *B. cinerea* alongside other fungi and oomycetes commonly associated with tomato crops demonstrated that the developed qcLAMP assay is highly target-specific. The assay also exhibited high diagnostic sensitivity (97.96%) across samples spanning a wide range of *B. cinerea* DNA concentrations, and a high accuracy rate (98.70%), defined as the percentage of correctly identified samples. Regarding analytical sensitivity, the limit of detection (LoD) of the qcLAMP assay was at least two orders of magnitude lower than that of conventional PCR, which served as the gold standard in this study (1 pg vs. 1 ng to 100 pg of genomic DNA per reaction, respectively). Notably, our conventional PCR results strongly align with and validate the findings of Fan et al. [3], whose protocol was adopted here, who reported a detection limit of 400 pg of DNA per reaction for *B. cinerea*. Compared to other LAMP-based assays targeting *B. cinerea*, the LoD of our developed assay was lower than 6.5 pg reported by Tomlinson et al. [6] and the 0.1 ng/μL reported by Si Ammour et al. [7], while matching the 10^−3^ ng/μL threshold reported by Duan et al. [28]. Furthermore, the qcLAMP assay exhibited a lower detection limit than the real-time PCR assay (6.25 pg) developed by Gachon and Saindrenan [5], and an equal sensitivity to the TaqMan real-time PCR assay (1 pg) by Mehli et al. [29]. However, it displayed a higher detection limit compared to the highly sensitive real-time PCR assay (20 fg) developed by Belen Suarez et al. [4]. Taken together, these findings indicate that the developed qcLAMP assay for *B. cinerea* demonstrates a remarkably high analytical sensitivity, often outperforming or matching alternative methods that require sophisticated bench-top equipment.

Regarding the detection of *B. cinerea* infective propagules (spores), the analytical sensitivity of the developed qcLAMP method was sufficient to detect consistently the lowest spore density (10^2^ *B. cinerea* spores) capable of inducing symptoms on artificially inoculated tomato leaf discs. The assay successfully detected this minimal inoculum load in both purified and crude DNA samples, highlighting its potential to identify *B. cinerea* on plant surface even when present below the critical effective inoculum threshold. In contrast, conventional PCR required an inoculum load at least two orders of magnitude higher (no fewer than 10^4^ spores) and succeeded only in purified DNA samples, failing to amplify crude extracts consistently across all spore concentrations. Although our observed detection limit is 10-fold higher than the 10^1^ spores detected via real-time (TaqMan) PCR by Belen Suarez et al. [4], the latter method carries inherent disadvantages, including mandatory sample purification, prolonged processing times, high operational costs, and the need for specialized personnel. Consequently, these operational constraints underscore the practical superiority and field-readiness of the developed qcLAMP assay.

*Botrytis cinerea* frequently establishes latent or asymptomatic infections within plant tissues, which transition into symptomatic outbreaks often triggered by shifting environmental conditions or host physiological changes [1,4]. Consequently, rapid and accurate detection of the pathogen is important to prevent its introduction into non-endemic areas and to mitigate economic losses in agricultural production [28]. In this study, the developed qcLAMP assay successfully detected *B. cinerea* immediately following spore deposition on leaf tissues (0 h post-inoculation) using both pure and crude DNA extracts from in vivo bioassays. This underscores its potential as a robust tool for diagnosing latent infections. In contrast, conventional PCR achieved detection only in pure DNA samples from leaves at 2 days post-inoculation (dpi), and in crude extracts at 3 dpi, when symptoms had already manifested. Although early, pre-symptomatic detection and quantification of *B. cinerea* has been previously demonstrated in pure DNA samples via real-time TaqMan PCR [4], it has not yet been reported using LAMP-based assays with crude plant extracts. Nevertheless, LAMP methodologies have successfully enabled early, on-site diagnostics for other plant pathogens using crude extracts, such as detecting latent *Phytophthora infestans* infections in potato leaves [30]. Similarly, recent advancements in micro-platforms and quantitative isothermal amplification have revolutionized the on-site detection of viral pathogens and viroids. For instance, colorimetric and barcoded RT-LAMP workflows have been successfully implemented for the rapid, extraction-free detection of Apple Stem Grooving Virus and Apple Scar Skin Viroid [25,26]. Furthermore, recent literature highlights the expanding versality of field-portable LAMP variants across a broader spectrum of phytosanitary applications. This is demonstrated both by battery-operated colorimetric RT-LAMP assays targeting devastating viral threats in solanaceous crops, such as Tomato Chlorotic Spot Virus [31], and by robust, duplexed assays developed for the in-field detection of major bacterial vascular diseases in fruit crops [32]. Much like our developed qcLAMP workflow for *B. cinerea*, these field-ready diagnostic configurations leverage simplified, inhibitor-tolerant sample preparation methods and portable optical readers to successfully bridge the gap between benchtop molecular screening and immediate epidemiological field interventions. This highlights the versatility of portable systems like the PEBBLE^®^ in achieving high analytical sensitivity outside the laboratory across diverse pathogen types, bridging the gap between fungal epidemiology and broad-spectrum phytosanitary screening.

The high tolerance of LAMP to reaction inhibitors facilitates its integration with simplified nucleic acid extraction protocols [33]. In the present study, the developed qcLAMP assay was coupled with a rapid, distilled water-based extraction method. This approach streamlined crude sample preparation by eliminating the need for extraction buffers and subsequent dilutions, thereby enhancing suitability for direct on-site applications. Nevertheless, across all independent experiments, purified DNA samples consistently yielded lower time-to-positive values compared to crude extracts. This possibly indicates that despite the assay’s robustness, host plant inhibitors can still affect the efficiency of the qcLAMP reaction. Notably, such differentiation in time-to-positive values between purified DNA and crude extract samples has not been previously reported for the LAMP assay.

Tomlinson et al. [6] emphasized that establishing the utility of a LAMP assay within a specific testing scenario requires evaluating naturally infected samples alongside symptom progression and existing diagnostic methods. In this regard, the efficiency of the developed qcLAMP assay was validated using purified DNA and crude extracts from asymptomatic and naturally infected tomato samples collected from a commercial greenhouse, and subsequently compared with conventional PCR. The results confirmed the findings of the in vivo experiments, demonstrating the superiority of the qcLAMP method across both template types (purified DNA and crude extracts) and disease status (symptomatic and asymptomatic). Specifically, for symptomatic leaves, the detection rates of qcLAMP and conventional PCR were 100% and 95% in purified DNA, and 100% and 0% in crude extracts, respectively. Furthermore, only the qcLAMP assay successfully detected the pathogen in asymptomatic tissues, yielding detection rates of 10% in purified DNA and 45% in crude extracts. Rather than indicating false positives, the positive signals in these apparently healthy samples can be attributed to latent or pre-symptomatic fungal infections. A current limitation of this study is that cross-reactivity against closely related *Botrytis* species (e.g., *B. fabae*, *B. squamosa*) was not explicitly evaluated herein. However, the IGS-targeting primers utilized in this workflow have been previously demonstrated to be highly specific to *B. cinerea* by Tomlinson et al. [6]. Similarly, Si Ammour et al. [7] reported that LAMP-based detection of *B. cinerea* in non-inoculated, field-collected bunch trash samples was due to natural colonization in the vineyard prior to collection. Consequently, the qcLAMP assay described herein represents a robust and reliable tool for the on-site detection of *B. cinerea* in tomato tissues. This contrasts with previous studies where LAMP method failed to reliably detect the same pathogen in field samples [28], highlighting the vital importance of validating novel diagnostic protocols using real-world agricultural samples.

It is worth highlighting the specific scientific contribution and novelty of our developed qcLAMP assay compared to existing molecular diagnostics for *B. cinerea*. While previous studies, including Tomlinson et al. [6], developed standard LAMP assays, they often relied on labor-intensive DNA purification steps, commercial extraction kits, or bench-top laboratory equipment, limiting their true field applicability. In contrast, although our workflow incorporates the primer sequences from Tomlinson et al. [6], it establishes a distinct diagnostic setup. Rather than reproducing the original experimental methodology, these primers were integrated into two novel technologies developed by BIOPIX DNA TECHNOLOGY: the Universal Mastermix, comprising a proprietary pre-optimized amplification formulation, and the portable PEBBLE^®^ diagnostic platform for real-time quantitative colorimetric monitoring. By adjusting the primer concentrations to this specific colorimetric formulation, we successfully adapted the assay to a completely different reaction chemistry and detection configuration. Consequently, this methodology transitions from a laboratory assay to a standalone, field-ready diagnostic platform that achieves an outstanding limit of detection (1 pg of genomic DNA and 10^2^ spores) directly from a crude water extract without any chemical buffers, centrifugation, or complex pipetting steps. Furthermore, a major scientific contribution of the present study is the comprehensive validation of the assays, using real field samples with natural infections. The unique capability of this point-of-care system to consistently detect laten, asymptomatic infections in 45% of real-world commercial greenhouse samples, directly within the farming environment where conventional PCR completely fails, demonstrates its high diagnostic power, clinical accuracy and distinct practical value for immediate, actionable epidemiological screening.

In view of all the above, the data presented in this study suggest that the developed qcLAMP method can serve as a rapid, reliable, and user-friendly tool for the early detection of *B. cinerea* in the field. By enabling prompt assessment of inoculum pressure, this assay facilitates timely management interventions and improves the overall control of grey mould in tomato crops. To fully maximize the potential of this approach, future efforts must focus on establishing robust sampling strategies that align with the biology, spatial distribution, and infection dynamics of the pathogen. Consequently, further research is needed to develop and optimize tailored sampling and assessment protocols coupled with the qcLAMP assay, ultimately leading to a more effective and sustainable disease management framework. Our results also demonstrated that the high sensitivity of the qcLAMP assay highlights the value of replicate measurements and appropriate quality-control procedures to support accurate interpretation of individual results under field conditions, particularly for early detection in asymptomatic tissues.

## 4. Materials and Methods

### 4.1. qcLAMP Primers and Assay Specifications

The intergenic spacer (IGS) region of the nuclear ribosomal DNA (*rDNA*) was selected as the target sequence for the LAMP assay. The *rDNA* region offers higher efficacy than alternative targets, while the IGS region provides greater sequence variation than the internal transcribed spacer (*ITS*) region, facilitating the identification of closely related species [34]. Moreover, the IGS region ensures high sensitivity and specificity [4] and occurs in multiple copies within the genome. A set of six primers targeting the IGS region of the *B. cinerea* ribosomal cistron was adopted from Tomlinson et al. [6], as presented in Table 4. Importantly, the concentrations of the six LAMP primers were specifically adjusted and optimized to match the colorimetric chemistry of the Universal Mastermix (BIOPIX-T), ensuring full compatibility with the amplification chemistry and the PEBBLE^®^ detection system.

All LAMP reactions were performed in the portable PEBBLE^®^ qcLAMP device with the use of the master mix from the same provider (BIOPIX-T, Heraklion, Crete, Greece). The PEBBLE^®^ platform is an integrated diagnostic system that enables nucleic acid amplification and real-time monitoring via quantitative colorimetric LAMP (qcLAMP). The device maintains the samples at the optimal reaction temperature while monitoring real-time color transitions of the Hydroxy Naphthol Blue (HNB) indicator during LAMP amplification, using an integrated LED illumination system and a digital camera. The final volume of the LAMP reaction was 25 μL and contained 12.5 μL 2x Universal Mastermix (BIOPIX-T), 2.5 μL 10× primer mix (BcF3 2 μM, BcB3 2 μM, BcFIP 18 μM, BcBIP 18 μM, BcLF 6 μM, BcLB 6 μM), 0.4 μL HNB 10 mM, 7.6 μL H2O and 2 μL of the template (template’s concentration and type varies depending on the experiment). To avoid evaporation and contamination in the PEBBLE device, 15 μL of mineral oil (included in BIOPIX-T kit) was added in each reaction mixture. The reactions were incubated in the PEBBLE at 78 °C for 30 min.

### 4.2. PCR Amplification

Conventional PCR amplification was employed as the gold standard method for *B. cinerea* detection in both pure DNA samples and crude extracts. The target DNA region of *B. cinerea* was amplified with the use of the primer set Bc-f (5′-CAGGAAACACTTTTGGGGATA-3′) and Bc-r (5′-GAGGGACAAGAAAATCGACTAA-3′) generating a 327-bp-long DNA fragment [3]. All PCR assays were carried out in an FG-TC01 FastGene^®^ Gradient thermocycler (NIPPON Genetics EUROPE), by the use of BK 1003 KAPA Taq PCR kit (KAPABIOSYSTEMS, Wilmington, Massachusetts, USA). PCR performance included an initial denaturation at 94 °C for 3 min; followed by 27 cycles of 30 s of denaturation at 94 °C, 30 s of annealing at 51 °C, and 60 s of extension at 72 °C; and a final extension step at 72 °C for 10 min. Amplification products were resolved on 1.5% (*w*/*v*) agarose gel in 1× TAE buffer, stained with Midori Green Advance and visualized under UV light.

### 4.3. Fungal/Oomycete Isolates and DNA Extraction

To evaluate the LAMP primers, seven representative isolates of *B. cinerea*, originated from greenhouse tomato (codes B1.1, B1.12, B14.9, B16.1, B22.7), cucumber (code B11.1) and pepper (code B13.11) crops, with typical grey mold symptoms and signs were employed [35]. The isolates were grown on potato dextrose agar (PDA) at 24 °C for 7 days in the dark, their mycelium was scraped from the surface of the culture using a sterilized scalpel, and their DNA was extracted according to Cary et al. [36]. In addition, to check primers’ specificity towards *B. cinerea*, DNA from cultures of other pathogens affecting tomato like the fungi *Verticillium dahliae* (isolate 999-1), *Alternaria* sp., (isolate MARKT2) and the oomycetes *Phytophthora infestans* (isolate 90128) and *P. nicotianae* (isolate CHATZ1), along with total DNA extracted from tomato leaves naturally infected by *Leveillula taurica*, were used. Moreover, DNA extracted from healthy tomato (hybrid Belladona F1) seedlings grown under controlled conditions at 23 ± 2 °C (with a 12 h light and dark cycle) served as the negative controls in the experiments. Total DNA from plant tissues was extracted based on the Cetyltetramethyl ammonium bromide (CTAB) method [37] with slight modifications, according to Soultatos et al. [38]. Purity and quantity of DNA were determined using a Q5000 UV-Vis Spectrophotometer (Quawell, San Jose, CA, USA). Final concentration of DNA samples was adjusted to 100 ng μL^−1^ and stored at −20 °C until use.

### 4.4. Determination of Limit of Detection, Sensitivity, Specificity and Accuracy

For the determination of the limit of detection (LoD) of the qcLAMP assay, a 10-fold serial dilution of genomic DNA extracted from *B. cinerea* (isolate B14.9) was prepared, with concentrations ranging from 100 ng/μL (corresponding to approximately 2 × 10^6^ genome copies/μL) down to 1 fg/μL (≈0.02 genome copies/μL). The same dilution series was also evaluated using conventional PCR, for comparison purposes. The LoD was defined as the lowest concentration of *B. cinerea* DNA yielded consistent amplification. To determine sensitivity, specificity and accuracy, genomic DNA extracted from seven distinct *B. cinerea* isolates was adjusted to the following concentrations: 10 ng/μL (≈2 × 10^5^ genome copies/μL), 4 ng/μL (≈8 × 10^4^ genome copies/μL), 1 ng/μL (≈2 × 10^4^ genome copies/μL), 100 pg/μL (≈2 × 10^3^ genome copies/μL), 10 pg/μL (≈2 × 10^2^ genome copies/μL), 5 pg/μL (≈100 genome copies/μL), 2.5 pg/μL (≈50 genome copies/μL), 1 pg/μL (≈20 genome copies/μL). A total of 77 DNA templates were analyzed, comprising 49 positive and 28 negative samples. To evaluate assay sensitivity, the 49 *B. cinerea*-positive DNA samples, spanning variable concentrations from 10 ng/μL to 1 pg/μL were tested, and assay sensitivity was estimated as the percentage of true positive results. To assess specificity, DNA samples from 5 non-target tomato pathogens and from a healthy tomato seedling were tested at a standard concentration of 1 ng/μL. Finally, diagnostic accuracy was calculated as the percentage of correctly identified samples (both true positives and true negatives) relative to the total number of tested templates.

### 4.5. Preparation of Crude Plant Extracts

To develop an easy-to-use, extraction-free sampling protocol, two crude extraction methods were evaluated. For the first method, approximately 0.5 cm^2^ of leaf tissue was collected and placed into 1.5 mL tube containing 0.5 mL TPS buffer (100 mM Tris-HCl, 1 M KCl, 10 mM EDTA, pH 8). The tissue was ground using a plastic pestle for approximately 30 s. Subsequently, a 20 μL aliquot of the supernatant was transferred into 80 μL of distilled H_2_O (dH_2_O), and 2 μL of the diluted plant extract was used as a template for the LAMP assay. To further simplify the sample preparation and eliminate the need for buffers and subsequent dilutions, an alternative protocol replacing the TPS buffer with water was evaluated. The water-based simplified protocol was performed as follows: approximately 0.5 cm^2^ of leaf tissue was placed into a 1.5 mL tube containing 1 mL dH_2_O and ground with a plastic pestle for about 15 s; then, 2 μL of the resulting plant extract was used directly as a template for the LAMP assay. To validate both methods, leaf samples from three *B. cinerea*-infected and three pathogen-free tomato plants were examined, with three technical replicates conducted per sample for each protocol. For verification and comparison reasons, an equal amount of leaf tissue from the same samples was subjected to standard DNA extraction using the CTAB method [37]; the resulting purified DNA samples were analyzed using both the newly developed LAMP assay and conventional PCR [3] to confirm the accuracy of the pathogen detection results.

### 4.6. Artificial Inoculation of Tomato Leaf Discs with Gradient Concentration of B. cinerea Conidial Suspension

To determine the inoculum density threshold required to induce grey mould symptoms on tomato leaves, a leaf-disc pathogenicity assay [4] was performed. Healthy tomato seedlings (hybrid belladonna F1) at the 5-true-leaf stage, grown under controlled conditions were used, and 10-mm-diameter leaf discs were excised from their lower leaves. For *B. cinerea* (isolate B14.9) inoculation, a conidial suspension was prepared by transferring four 25-mm-diameter mycelial plugs from a 2-week-old PDA culture into a 50 mL Falcon tube containing 30 mL of sterilized dH_2_O, followed by vortexing for 30 s. The suspension was filtered through two layers of sterilized gauze, and the conidial concentration was determined using a haemocytometer. Dilutions were made to obtain final concentrations of 10^6^, 10^5^, 10^4^ and 10^3^ spores mL^−1^. Artificial inoculation was conducted by depositing a single 10-μL drop of each suspension onto the center of each leaf disc (corresponding to 10^4^, 10^3^, 10^2^ and 10^1^ spores per disc, respectively), whereas control discs were mock-inoculated with 10 μL of sterilized distilled water (0 spores per disc). Prior to drop deposition, the inoculation site was lightly wounded using sterilized forceps. Artificially and mock-inoculated discs were then placed onto plastic laboratory racks and enclosed in transparent plastic boxes (50 × 38 × 36 cm in size). Moist tissue paper was placed at the bottom of each box to maintain high humidity (90% RH). The boxes were incubated in a growth chamber at 20 ± 1 °C under fluorescent lights with a 12-h photoperiod for 7 days. Ten replicate discs were used per treatment. Disease symptoms were monitored daily by measuring the lesion diameter (rotted area and/or area covered by fungal mycelium) on each disc. At the end of the experiment, all discs were examined visually and stereoscopically to detect the presence of fungal fructifications. Additionally, microscopic samples were prepared and observed under a light microscope to identify *B. cinerea* and verify successful inoculation.

### 4.7. Evaluation of the qcLAMP Capacity to Detect B. cinerea Propagules in Crude Extracts

*Botrytis cinerea* is primarily dispersed by airborne inoculum (conidia) that lands onto plant surface and infects susceptible tissues [1]. To evaluate the capacity of the developed qcLAMP assay to detect infective propagules present on the tissue surface, the leaf-disc assay was employed using various inoculum levels, following the procedure described above. Specifically, a 10-μL drop of conidial suspensions was deposited onto each leaf disc to achieve final inoculum loads of 10^5^, 10^4^, 10^3^, 10^2^, 10^1^, 1 and 0 spores per disc. The discs were kept under ambient conditions for 1 h to allow the deposited drops to air-dry. Then, individual discs (one disc per sample) were collected and subjected either to standard DNA extraction using the CTAB method [37], or to crude extraction using the water-based protocol developed in this study. The resulting purified DNA and crude extract samples were analyzed using both the newly developed qcLAMP assay and conventional PCR [3]. Three independent replicate discs were used per treatment (inoculum level), extraction method (standard DNA extraction or crude extraction) and amplification assay (conventional PCR or qcLAMP).

### 4.8. Evaluation of the qcLAMP Assay for the Presymptomatic Detection of B. cinerea

The potential of the developed qcLAMP assay to detect *B. cinerea* prior to symptom development was evaluated using the leaf-disc assay. Tomato leaf discs were drop-inoculated with 10^3^ spores of *B. cinerea* per disc (designated as TB1, TB2 and TB3), whereas mock-inoculated discs served as controls (designated as TC1, TC2 and TC3). Both inoculated and mock-inoculated discs were placed onto racks, enclosed in plastic boxes, and incubated under the controlled conditions described above. Individual discs (one disc per sample) were collected at 0, 6, and 12 h post-inoculation (hpi), as well as at 1, 2, 3, 4, and 5 days post-inoculation (dpi). Prior to sampling, each disc was visually evaluated for symptom development. The collected discs were subjected either to standard DNA extraction using the CTAB method [37] or to crude extraction using the water-based protocol. The resulting purified DNA and crude extract samples were analyzed using both the newly developed qcLAMP assay and conventional PCR. Three independent replicate discs were used per sampling time, extraction method, and amplification assay. Concurrently, an additional 10 artificially inoculated and 10 mock-inoculated discs were maintained under the same controlled conditions, and served as external reference to monitor disease progress and verify inoculation success. Disease symptoms were monitored by measuring the lesion diameter on each disc at each sampling point.

### 4.9. Validation of the Efficiency of the Developed Method in Field Samples

To validate the efficiency of the developed qcLAMP method under field conditions, a sampling survey was conducted in March 2024, in an organic, semi-hydroponic tomato greenhouse (hybrid Elpida F1) located in Ampelouzos (Heraklion, Crete, Greece), which exhibited a severe incidence of grey mould. A total of 20 apparently healthy (designated as TC1 to TC20) and 20 diseased tomato leaves displaying typical *B. cinerea* symptoms and signs (designated as TBC1 to TBC20) were collected. Samples were individually placed in plastic bags, kept in ice boxes, and immediately transferred to the laboratory for further analysis. Two individual leaflets from each leaf were selected and a 10-mm-diameter leaf disc was excised from each leaflet; one disc was subjected to standard DNA extraction using the CTAB method [37], while the other underwent crude extraction via the water-based protocol. The resulting purified DNA and crude extracts were analyzed using both the newly developed qcLAMP assay and conventional PCR. To prevent potential degradation in the crude extracts that could compromise conventional PCR efficiency, sample processing was strictly managed in the laboratory to ensure extract stability, thereby enabling an accurate comparison between the two assays.

### 4.10. Statistical Analysis

In artificial inoculation experiments using gradient concentrations of *B. cinerea* conidial suspensions, analysis of variance (ANOVA) was performed to evaluate the pathogenic effects of different inoculum loads on leaf discs. When a significant *F* test was obtained for treatments (*p ≤* 0.05), the data were subjected to means separation by Tukey’s HSD test, and standard errors of means were calculated. Moreover, in artificial inoculation for the presymptomatic detection of *B. cinerea* with the qcLAMP assay, data on lesion diameter between artificially inoculated and mock inoculated discs were analyzed by carrying out a two-sample *t* test (*p ≤* 0.05). All experiments were performed in triplicate. Time-to-positive results for the qcLAMP assay are presented as mean ± standard deviation (SD). Due to the clear qualitative nature (detected/not detected) of the majority of readouts, no additional complex statistical analyses were required.

## Figures and Tables

**Table 1:** Comparative evaluation of qcLAMP and conventional PCR assays using purified DNA (CTAB method) and crude extracts (dH_2_O-based method) from leaf discs inoculated with varying *B. cinerea* (isolate B.14.9) spore densities (infective propagules). Samples were collected 1 h post-inoculation, following the complete air-drying of the applied conidial drops.

Inoculum Propagules	Purified DNA Samples		Crude Extract Samples	
	PCR ^1^	qcLAMP ^2^	PCR ^1^	qcLAMP ^2^
10^5^ spores	+/+/+	n.a./n.a./n.a.	-/-/-	+ (20.8 min)/+ (15.3 min)/+ (22.3 min)
10^4^ spores	+/+/+	+ (16.8 min)/+ (13.3 min)/+ (16.2 min)	-/-/-	+ (22.2 min)/+ (19.2 min)/+ (23.3 min)
10^3^ spores	-/-/-	+ (15.0 min)/+ (14.3 min)/+ (14.8 min)	-/-/-	+ (22.5 min)/+ (20.5 min)/+ (24.2 min)
10^2^ spores	-/-/-	+ (23.7 min)/+ (13.7 min)/+ (15.5 min)	-/-/-	+ (34.2 min)/+ (30.3 min)/-
10^1^ spores	-/-/-	-/-/-	-/-/-	-/-/-
1 spore	-/-/-	+ (17.0 min)/-/+ (14.0 min)	-/-/-	-/-/-
0 spores	-/-/-	-/-/-	-/-/-	-/-/-

^1^ The symbols ‘+’ and ‘-’ represent positive and negative detection, respectively, in conventional PCR assays. Results are presented sequentially for each independent replicate (1st, 2nd, and 3rd rep.). ^2^ In qcLAMP assays, ‘+’ and ‘-’ indicate positive and negative detection, respectively, followed by the time-to-positive value (in minutes); ‘n.a.’ indicates that the respective samples were not analyzed with the qcLAMP assay. Results are presented sequentially for each independent replicate (1st, 2nd, and 3rd rep.).

**Table 2:** Comparative evaluation of qcLAMP and conventional PCR for the pre-symptomatic detection of *B. cinerea* (isolate B.14.9). Assays were performed using purified DNA (CTAB method) and crude extracts (dH_2_O-based method) from leaf discs inoculated with 10^3^ spores per disc, sampled at 0, 6, and 12 h post-inoculation (hpi) as well as at 1, 2, 3, 4 and 5 days post-inoculation (dpi).

Treatment	Time	Purified DNA Samples		Crude Extract Samples	
		PCR ^1^	qcLAMP ^2^	PCR ^1^	qcLAMP ^2^
Mock-inoculated	0 hpi	-/-/- (0 mm)	-/-/-	-/-/- (0 mm)	-/-/-
	6 hpi	-/-/- (0 mm)	-/-/-	-/-/- (0 mm)	-/-/-
	12 hpi	-/-/- (0 mm)	+ (20.3 min)/-/-	-/-/- (0 mm)	-/-/-
	1 dpi	-/-/- (0 mm)	-/-/-	-/-/- (0 mm)	-/-/-
	2 dpi	-/-/- (0 mm)	-/-/+ (17.8 min)	-/-/- (0 mm)	-/-/-
	3 dpi	-/-/- (0 mm)	-/+ (16.7 min)/-	-/-/- (0 mm)	-/-/-
	4 dpi	-/-/- (0 mm)	n.a./n.a./n.a.	-/-/- (0 mm)	-/-/-
	5 dpi	-/-/- (0 mm)	n.a./n.a./n.a.	-/-/- (0 mm)	-/-/-
*B. cinerea*-inoculated	0 hpi	-/-/- (0 mm)	+ (15.7 min)/+ (14.8 min)/+ (16.5 min)	-/-/- (0 mm)	+ (28.0 min)/+ (27.0 min)/+ (25.0 min)
	6 hpi	-/-/- (0 mm)	+ (15.5 min)/+ (14.3 min)/+ (15.7 min)	-/-/- (0 mm)	+ (21.0 min)/+ (24.5 min)/+ (21.5 min)
	12 hpi	-/-/- (0 mm)	+ (14.8 min)/+ (12.7 min)/+ (16.2 min)	-/-/- (0 mm)	+ (16.8 min)/+ (18.7 min)/+ (16.7 min)
	1 dpi	-/-/- (2/0/2 mm)	+ (13.3 min)/+ (11.5 min)/+ (15.2 min)	-/-/- (0/2/1 mm)	+ (15.5 min)/+ (15.8 min)/+ (14.5 min)
	2 dpi	+/+/+ (2.5/3/5 mm)	+ (13.8 min)/+ (11.0 min)/+ (12.2 min)	-/-/- (4/4/7 mm)	+ (15.0 min)/+ (13.8 min)/+ (14.8 min)
	3 dpi	+/+/+ (7/9/9.5 mm)	+ (12.7 min)/+ (12.0 min)/+ (13.2 min)	+/+/+ (10/10/9.5 mm)	+ (13.0 min)/+ (11.3 min)/+ (12.0 min)
	4 dpi	+/+/+ (10/10/10 mm)	n.a./n.a./n.a.	-/-/- (10/10/10 mm)	+ (15.2 min)/+ (15.8 min)/+ (15.0 min)
	5 dpi	+/+/+ (10/10/10 mm)	n.a./n.a./n.a.	-/-/- (10/10/10 mm)	+ (14.7 min)/+ (13.8 min)/+ (14.3 min)

^1^ The symbols ‘+’ and ‘-’ represent positive and negative detection, respectively, in conventional PCR assays, followed by the lesion diameter (mm) measured on the sampled disc surface. Results are presented sequentially for each independent replicate (1st, 2nd, and 3rd rep.). ^2^ In qcLAMP assays, ‘+’ and ‘-’ indicate positive and negative detection, respectively, followed by the time-to-positive value (in minutes); ‘n.a.’ indicates that the respective samples were not analyzed with the qcLAMP assay. Results are presented sequentially for each independent replicate (1st, 2nd, and 3rd rep.).

**Table 3:** Detection of *B. cinerea* in symptomatic and asymptomatic tomato leaf samples collected from a commercial greenhouse (Ampelouzos, Heraklion, Crete, Greece), using qcLAMP and conventional PCR. Assays were performed using purified DNA (CTAB method) and crude extracts (dH_2_O-based method) from leaf tissues.

Disease Status	Sample Code	Purified DNA Samples		Crude Extract Samples	
		PCR ^1^	qcLAMP ^2^	PCR ^1^	qcLAMP ^2^
Apparently healthy	TC1	-	+/15.5 min	-	-
	TC2	-	-	-	+/27.8 min
	TC3	-	-	-	+/22.7 min
	TC4	-	-	-	+/22.3 min
	TC5	-	-	-	-
	TC6	-	-	-	-
	TC7	-	-	-	-
	TC8	-	-	-	+/27.5 min
	TC9	-	-	-	-
	TC10	-	-	-	+/27.5 min
	TC11	-	-	-	-
	TC12	-	-	-	-
	TC13	-	+/23.2 min	-	-
	TC14	-	-	-	-
	TC15	-	-	-	+/21.7 min
	TC16	-	-	-	+/29.8 min
	TC17	-	-	-	-
	TC18	-	-	-	+/30.8 min
	TC19	-	-	-	+/22.5 min
	TC20	-	-	-	-
Diseased	TBC1	+	n.a.	-	+/17.7 min
	TBC2	+	n.a.	-	+/17.5 min
	TBC3	+	n.a.	-	+/11.0 min
	TBC4	+	n.a.	-	+/16.0 min
	TBC5	+	+/11.7 min	-	+/17.3 min
	TBC6	+	n.a.	-	+/19.2 min
	TBC7	+	n.a.	-	+/17.5 min
	TBC8	+	n.a.	-	+/16.0 min
	TBC9	+	n.a.	-	+/14.8 min
	TBC10	-	+/14.8 min	-	+/25.7 min
	TBC11	+	n.a.	-	+/15.8 min
	TBC12	+	n.a.	-	+/16.8 min
	TBC13	+	n.a.	-	+/17.5 min
	TBC14	+	n.a.	-	+/16.7 min
	TBC15	+	n.a.	-	+/18.2 min
	TBC16	+	n.a.	-	+/19.2 min
	TBC17	+	n.a.	-	+/16.7 min
	TBC18	+	n.a.	-	+/17.3 min
	TBC19	+	+/14.9 min	-	+/15.7 min
	TBC20	+	n.a.	-	+/17.0 min

^1^ The symbols ‘+’ and ‘-’ represent positive and negative detection of the target pathogen, respectively, in conventional PCR assays. ^2^ In qcLAMP assays, ‘+’ and ‘-’ indicate positive and negative detection, respectively, followed by the time-to-positive value (in minutes); ‘n.a.’ indicates that the respective samples were not analyzed with the qcLAMP assay.

**Table 4:** Loop-mediated amplification (LAMP) primers used in this study (sequences originally described by Tomlinson et al. [6]).

Primer	Sequence (5′→3′)
BcF3	TCGGAGTGTCCTAGGAATGC
BcB3	TGAGATGGCCAACTCTCAGA
BcFIP	GCCTGCTCACCGGTAGTAGTGTGTGAGCCCTTGGTCTAAAGC
BcBIP	GCAGAATCTGTCCCCGGTGAGCGGGAGCAACAATTAATCGC
BcLF	TGGGGTTAACTAGTCACCTATACG
BcLB	AGGTCACCTTGCAATGAGTGGA

## Data Availability

The data presented in this study are available within the article. Additional raw data that support the findings are available from the authors upon reasonable request, subject to confidentiality restrictions.

## Abbreviations

The following abbreviations are used in this manuscript:

qcLAMP	quantitative colorimetric loop-mediated isothermal amplification
LoD	limit of detection
PCR	polymerase chain reaction
CTAB	Cetyltetramethyl ammonium bromide
HNB	Hydroxy Naphthol Blue
